# On the Remedial and Anæsthetic Uses of Intense Cold

**Published:** 1855-01

**Authors:** James Arnott

**Affiliations:** London


### SELECTIONS.

*On the Remedial and Anæsthetic uses of intense cold.* By JAMES ARNOTT, M.D., London.

Although the subjeccts of the remedial efficacy of congelation and local anæsthesia from cold have been for some years before the public, they are as yet but little understood and appreciated. This has resulted, partly from their having been imperfectly explained, in consequence of the publications respecting them being severally incomplete, and partly from the strength of the prejudice against extreme cold. Dr. Rowley, who, in his attack on cowpox, declared that the accounts which he had heard of the terrible effects of communicating the "cruel and beastly" disease were enough to "freeze the soul," was probably not more horror-stricken than some have been by the proposal to freeze the body; and the introducer of vaccination was hardly more abused than the proposer of congelation has been. It is in the hope that this prejudice may be thereby abated, and the subject rendered better understood, that the following brief statement is published. Even in France, where both the remedial and anæsthetic uses of intense cold have been turned to account for some time by M. Velpeau, and other leading practitioners, there is still much doubt about the best mode of applying the agent. In a paper in the Bulletin de Therapeutique, of the 15th ult., M. Richet, Surgeon of the Hospital Saint Antoine in Paris, reports thirteen operations in which local anæsthesia had been produced by the very imperfect means of the quick evaporation of ether.

As no remedy has been longer in use, and few are more valued than the local application of moderate degrees of cold, or a temperature ranging from that of dissolving ice to about 70 degrees of Fahrenheit, it may at first appear singular, that a greater or more powerful remedial effect should not have been sought by increasing the dose of the agent, or employing a lower temperature, in the same manner as we have sought and found much greater remedial benefit in many cases by using mercury, antimony, quinine, and other drugs, in larger doses than had been customary. The reason is that medical men were under a most erroneous impression respecting the effects of very low temperatures on the body. Because a temperature of zero stops the circulation, and because the vitality of a part has been lost by its *long-continued* congelation, whether caused by exposure to severe cold in winter, or by the incautious use of ice in hernia and other diseases, it was hastily and erroneously inferred that there was danger of loss of vitality from *short-continued* congelation. The mistake would not be greater to infer from the fact, because a long-continued stoppage of the circulation through a limb from an improper application of a bandage has occasioned gangrene, that it would be dangerous to use the tourniquet in operations.

The correction of this error will be deemed of no little importance when it is considered that in short-continued congelation, judiciously applied, we have an unfailing means of immediately arresting inflammation wherever it can be reached by the remedy; of not only giving speedy relief from pain in many diseases, but in consequence of the organic changes produced by it, of obviating the return of pain; and in malignant disease, of producing an amount of benefit much exceeding that yet accomplished by other means. Although much inferior in importance to these results, it is yet another great benefit conferred by intense cold, that the pain which would be otherwise caused by the greater number of surgical operations can be prevented by it with perfect safety; and not only can pain be prevented, but the inflammation proceeding from the surgeon's knife, that so often proves fatal, may also be obviated by the same means, and with almost equal certainty. It will be proper to consider the remedial and anæsthetic effects of intense cold separately; but before doing so, it is necessary to mention how this degree of cold is produced and applied, as well as to attempt an explanation of its mode of operation.

That degree of cold may be called intense which immediately benumbs the part to which it is applied, speedily stops the circulation through it, and congeals the adipose matter. I have usually produced these effects by placing what are termed frigorific mixtures either immediately in contact with the skin or mucuous membrane, by means of a net of thin guaze containing them, or by allowing them to act through thin bladders, or metallic vessels of appropriate form; but there are various other ways of effecting the same object, some of which are preferable for certain purposes. Substances passing rapidly from the solid to the fluid, or from the fluid to the æriform state, strongly abstract caloric from other bodies in contact with them; and substances, either solid fluid, or æriform, already sufficiently cooled by artificial means, may be placed in contact with the part; the first, as solid metallic balls of appropriate shape; the latter two, when forming strong currents. When cold is produced by the common frigorific mixture of ice and salt, and applied by means of a guaze bag or net, the following is a convenient mode of proceeding:—If the congelation is not to be extensive or long continued, a piece of ice of the size of a large orange will be sufficient. This is well pounded in a coarse cloth or bag, and the powder being placed upon a large sheet of paper, is thoroughly mixed, by means of a paper folder, with about half its weight of common salt. The mixture is then put into a net of about four inches diameter, and as soon as it begins to dissolve, it is ready to be applied. The net is not kept motionless on the part, but is frequently raised in order that fresh particles of the mixture may be brought in contact with the skin; and the water that escapes from it may be absorbed by a sponge, or allowed to fall into a basin placed underneath. If the surface to be acted upon is of small extent, a very thin and large copper spoon containing the mixture or a solid brass ball of about a pound weight, which has been immersed in ice and salt, will often answer and be a neater mode than the net.

The moment a guaze net, or a thin metallic vessel containing ice and salt, is applied to the skin, it is benumbed. There is hardly a sensation of cold produced, and no tingling or smarting. If the contact of the frigorific be continued a few seconds longer, the surface becomes suddenly white, in consequence, doubtless, of the arrest of the circulation; and this change of color is attended with a slight smarting like that produced by mustard. There is now complete anæsthesia, which, if the frigorific be allowed to act, another change is produced—the adipose matter under the skin is congealed, and the part becomes hard as well as white. The depth to which the benumbing influence of cold will extend depends upon a variety of circumstances, as the degree of cold, the duration of the application, the vascularity of the part, whether pressure is used or the circulation is suspended, &c., &c. After the usual application of cold for anæsthesia, the circulation soon returns to the part, and the skin assumes a red color which lasts for several hours. If the congelation has been considerable, there is now some smarting felt, unless the natural heat be more gradually restored by pouring cold water on the part, or by placing on it a little pounded ice, or a bladder containing iced water. If the application has not exceeded the first stages, there is no smarting and no necessity, therefore, for such precautions.

The redness produced does not, as might at first sight be supposed, indicate an inflammatory condition, but the very reverse. The tonicity of the small arteries appears to be lessened or suspended for a time, and instead of being inflamed, the part is rendered unsusceptible of inflammation. Parts cut after congelation heal by adhesion or the first intention more quickly than they otherwise would; and, as has already been said, we possess in this expedient a certain and prompt remedy for every inflammation accessible to its complete influence.

I. *Reemedial Uses of Intense Cold.*—The remedial qualities ef intense cold may be described as antiphlogistic, anodyne or sedative, and specific; and it is useful in the diseases for which other remedies possessing these qualities have been employed, viz., in inflammatory, painful or irritative, and malignant diseases. The circumstance which limits its application in these, is the impossibility of extending its influence beyond a certain extent or depth, although it is certain from its effects in deep-seated disease, that this influence, whether it be direct or sympathetic, is more extensive than would at first be supposed. It may be laid down as a rule, that in every case in which the local application of moderate degrees of cold has been found of service, the use of well regulated congelation would prove much more useful; and in those diseases of similar character, in whieh moderate cold has not been employed from the idea that their seat was beyond its reach, congelation might be tried with reasonable hope of success. Intense cold has this immense advantage over other powerful remedies of the same class, that may be used with impunity—if it does no good it will do no harm. Who will venture to affirm this of bleeding, mercury, antimony, opium, chloroform, arsenic? Neither in my own practice, nor (so far as I can learn) in the practice of others, has there been any untoward result from the use of congelation. Its action being confined to the diseased part, and not uselessly expended on the rest of the system, affords the explanation. Other topical remedies have much the same character for safety, but what other expedient of this class has a tenth part of the power of intense cold?

Instead of enumerating the diseases in which this agent has been employed according to the above cla sification, I shall mention, first, those in which it has been more or less successful; and second, those in which it might, reasoning from analogy, be tried with hope of advantage. In administering intense cold as a remedy, the common or a more powerful frigorific has been generally applied directly to the part, or with the intervention only of the thin gauze containing it; and the duration of the congelation has been from one to ten minutes.

In the spring of the year 1849, I requested the house-surgeon of Brighton dispensary to apprise me of every case of acute lumbago that came under his notice, and in all of these amounting to nine, I employed congelation with perfect and permanent success. The net containing the ice and salt was passed to and fro for five minutes, over a surface of about eight by four inches, the skin being blanched during the whole of this period. In only two or three cases was it necessary to apply the remedy twice. Several of the patients rose immediately afterwards from their beds to which they had long been confined. In most cases of chronic rheumatism, the remedy has been equally successful; and this, on account of the frequency of the disease, is one of its most valuable applications. Sciatica has generally yielded to it, but by no means so easily. In acute rheumatism, the local inflammation of the joints is, by this means, invariably and completely relieved, and that portion of the accompanying fever thence arising, is consequently removed. The disease, thus treated, will run a painless course of about a week's duration In no case, of about a dozen in which congelation was almost exclusively employed, was there extension of inflammation to the heart; and I am persuaded that the best plan of preventing this, is to subdue the inflammation of the joints from which it generally originates. I did not use the remedy in cases where the heart was already affected, though I have since learned that congelation is employed in the hospital at Vienna (where it was introduced some years ago by Dr. Waters of Chester,) as an application to the chest in rheumatic carditis. That this affection of the heart would occasionally occur during the treatment of acute rheumatism by congelation is very probable, because it often arises, as the same affection of the joint does, from a morbid condition of the blood over which the remedy can have no control; and that such an occurrence, in the present feeling on the subject, would be called metastassis from cold, is very certain; but I am convinced that it will yet be acknowledged, though probably after many years that this affection would be much decreased in frequency by the adoption of any means capable of quickly subduing the accompanying arthritis. When it is considered what an immense amount of eventual mischief arises from the organic disease of the heart that occurs under the common modes of treating rheumatic fever, to say nothing of the patient's present sufferings and tedious confinement, it is to be lamented that prejudice should oppose any means of greater promise. In rheumatic gout, the relief has been as marked from congelation as in lumbago. In ordinary inflammation of the joints it has also been exceedingly useful. Ophthalmia has been immediately cured by keeping the frigorfic in contact with the gently closed eye-lid for three or four minutes. Glandular inflammation in the neck and groin, yield to a high degree of cold with equal facility. I have been told that in orchitis, its beneficial operation is immediate; and I have little doubt that, from its closeness to the surface, the urethral inflammation causing orchitis, would be quickly suppressed.

Congelation has often at once converted an irritable into an healing ulcer, though sometimes the patient has complained of the pain of the operation; it is probable that had the salt in the mixture been prevented coming in contact with the irritable surface, this would have been in a great degree prevented. Certain acute inflammatory affections of the skin are equally under its influence, as erysipelas, eczema, impetigo. It has not often failed in purigo, but in only one case of psoriasis has it appeared to be of service. Painful nodes are at once relieved by this means, and the inflammation subdued. I have only used congelation in carbuncle as an anæsthetic previously to cutting it, but it is probable (judging from its effects in severe boils) that the incisions might have been dispensed with. It has been mentioned to me that severe cold has been employed with the same view in whitlow, of which it is certainly a perfect cure. The inflammation following sprains, contusions and other similar injuries is perfectly under its influence; and the same may be said of burns. In one of my publications on the subject, I have related the excellent and speedy effect of congelation in a case meningitis, and also in a case of peritonitis; I have not had the opportunity of trying it in other affections of this description. Headache of various kinds has at once yielded to the application, for a minute, of a frigorific over the painful part; and in neuralgia affecting the side, it has generally proved efficacious. In neuralgia attacking the face and other parts, it has often succeeded and often failed. If the seat of the disease be deep in the brain, little can be hoped from this remedy, although there are few obstinate cases of neuralgia in which it does not deserve a trial. Toothache is generally at once relieved by it if properly applied; and there is no remedy for the painful affection of the mouth caused by mercury comparable to congelation. A spoonful of dissolving ice and salt is repeatedly put into the mouth, until it becomes benumbed. In one case of severe scurvy of the gums, where I feared a loss of the teeth, extensive congelation of the gums immediately arrested the disease.

In many of the diseases just enumerated, the promptness of the cure is as remarkable as its certainty. In military and hospital practice this advantage is very prominent.

In cancer the effects of congealation have been various. From my own experience, and that of others, I think that in early stages and when from its size the tumor can be thoroughly brought under the influence of the remedy, it will be cured by it. In all stages the progress of cancer will be arrested or retarded, and the pain accompanying it assuaged. The difficulty in advanced cases is to cause a sufficient degree of heat and cold to pervade the tumor.—The French translator of a recent paper of mine on the snbject (L'Union Medicale for May,) thinks that the frequent occurrence of cysts in cancerous tumors may facilitate this. But if layer after layer is acted upon, it may be enough. In cancer of the womb the frigorific is applied by way of a speculum, and one stronger than ice and common salt will generally be required. The opinion of Dr. Hughes Bennett respecting the nature of cancer have much influenced the mode in which I have used congelation in its treatment. M. Velpeau states in his recent elaborate work on diseases of the breast, that he has employed long-continued congelation as a substitute for caustic in cancer; but of this effect of the agent I have no knowledge.

There are other diseases in the treatment of which severe cold would probably be very useful. It might be applied with such a hope to the spine in tetanus, or to the scalp in certain varieties of mania. After gun-shot and other severe wounds, it would prove a powerful preventive and cure of inflammation. Even in pleuritis and other deep-seated inflammation of the chest, as well as in various uterine affections, benefit might rationally be expected from it. In two cases of epidemic cholera, I administered a succession of draughts of a temperature of about 25° Fahrenheit, with apparently excellent effect; and I cannot doubt that the application of cold to the interior of the stomach, which as appears by the recently published report of the College of Physicians, is the only treatment of cholera which has been unanimously approved of, has not been carried far enough. If the irritation of the mucous membrane be considerable (as it must be, to account for the exhausting and fatal discharges,) the temperature of ice merely is not sufficient to subdue it.

II. *Anœsthetic Uses of severe Cold.*—As patients now expect to have every operation performed without pain, both they and their surgeons will be glad to have an easy and agreeable means of accomplishing this, in all the common operations, unaccompanied with the dangers of chloroform. What can be less troublesome in opening an abscess, for instance, or making a cutaneous incision, than touching the skin for a moment with a brass ball that has been immersed for a few minutes in ice and salt, or a thin spoon filled with such a mixture? It is true, that in deep-seated operations such a means can only suspend the sensibility of the skin; but it is the incision of the skin which constitutes the most painful part of every operation and if this be benumbed, a smaller, and consequently less hazardous dose of ether or chloroform than has usually been administered, would be enough to remove the sensibility of the other tissues. These deep-seated operations, however, constitute a small minority, and if the list recorded deaths from etherization be referred to (now amounting to more than fifty) it will be found that in three-fourths of the number, complete anæsthesia might have been produced with perfect safety by cold.

M. Velpeau, who introduced anæsthesia from cold into France, has, in a lecture on the subject recently reported in the Gazette des Hospitaux, expressed the doubt, whether in some operations, the hardening of tissues might not prevent their being cut with ease. I have not found this to be the case, nor does he himself allude to this disadvantage, when, in his work on diseases of the breast, he mentions that he has excised tumors after anæsthesia from cold.

The fear of re-action I have already adverted to in in the prefatory observations. Instead of re-action by being produced, the anæsthetic is a preventive of inflammation from the wound; and were it used for this purpose alone, it would be invaluable.

Local anæsthesia from cold may, as has already been observed, be produced in a great variety of ways. Some of these may be applied so as to cause immediate congelation, but it is questionable whether the anæsthesia is not more extensive and lasting when more slowly caused. Such details, however, are unsuited to the general view of the subject intended by the present communication, which, I fear, has already exceeded its proper bounds.—*Edinburgh Monthly Jour. of Med. Science.*

